# Pleomorphous leiomyosarcoma of the mesocolon: a case report

**DOI:** 10.11604/pamj.2015.22.322.8050

**Published:** 2015-12-02

**Authors:** Saad Rifki Jai, Robleh Hassan Farah, Brahim Hamdaoui, Rachid Boufettal, Farid Chehab

**Affiliations:** 1 Service de Chirurgie Viscérale Aile 3, Centre Hospitalier Universitaire Ibn Rochd, Université Hassan II, Casablanca, Maroc

**Keywords:** Pleomorphous leiomyosarcoma, mesocolon, smooth muscle tumors

## Abstract

Leiomyosarcoma is a rare tumor of the smooth muscle, but relatively frequent in the stomach and the small intestine. The mesocolic site is rare. Globally, leimyosarcoma represents less than 0, 1% of the malignant tumors found in the colon and the anus. Because of the similarities with other digestive tumors, namely mesenchymatous or benign tumors of the smooth muscle, the diagnosis of a pleomorphic sarcoma remains difficult even at the histological stage. Surgery is the mainstay of the therapy. We report a case of leiomyosarcoma of the mesocolon and discuss about its main characteristics in the view of the current literature about this pathological condition.

## Introduction

Soft tissue sarcomas (STS) are an extremely diverse group of tumors occurring in every region of the human body and affecting all ages with significant morbidity and mortality. Soft tissue sarcomas include more than 35 different neoplasms derived from fat, muscle, nerve, fibrous, vascular, or deep skin tissue, and can be classified by their tissue of origin or their anatomical location [1]. The World Health Organization classifies liposarcomas into 5 histologic subtypes: welldifferentiated, dedifferentiated, myxoid, round cell, and pleomorphic sarcomas. Dedifferentiated histologic subtypes have a worse prognosis and an increased risk of local recurrence compared with well- differentiated liposarcomas [2]. Therefore, aggressive complete surgical resection of the tumor and adjacent organs is the mainstay of treatment for dedifferentiated liposarcomas [2]. We report here a case of pleomorphic leiomyosarcoma arising from the mesocolon and discuss its main characteristics and the management of this tumor along with a review of the literature.

## Patient and observation

A - 62-year- old female patient, who had past medical history of well treated pulmonary tuberculosis 6 years ago, that hospitalized because of a chronic abdominal pain for 10months. The patient reported weight loss about 6-kg during her illness. The physical examination found palpable per-umbilical mass (10 cm of diameter), hard and mobile relatively to the deeper and superficial neighboring structures. No lymph node or ascites was found. Laboratory exams: complete blood count was in normal range and tumor markers were negative.

**Figure 1:**
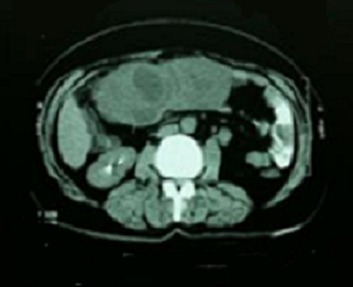
CT scan of the abdomen showing a round tissular mass with irregular edges with central necrotic areas enhancement in the peripheral zones

**Figure 2:**
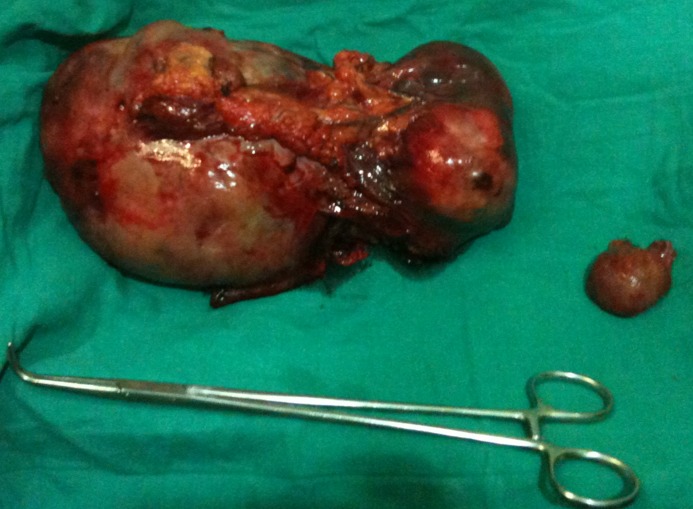
Removed specimen of the tumor and the adjacent part of the mesecolon

Computer tomography scanning of the abdomen showed a round tissular mass with irregular edges, which makes 14 cm in its great axis, with central necrotic areas enhancement in the peripheral zones (Figure 1). A primitive pelvic tumor was suspected. At laparotomy exploration, the surgeons noted a pink multiloblar tumor of the transverse mesocolon of 15 cm is his greatest axis, without any ascites or peritoneal carcinoid. The surgeons performed a resection of the tumor and the adjacent part of the mesocolon (Figure 2).

On macroscopically exam, the tumor was 15cm of diameter, firm with necrotic areas. Histopathological examination combined with immunohistochemical studies established the diagnosis of pleomorphous sarcoma of the mesocolon. The postoperative course was uneventful. No adjuvant therapy was instituted.

## Discussion

Pleomorphous sarcomas represent approximately 10 to 15% of the sarcoma of the smooth tissues of the adult [3]. The mean age at occurrence is about the 6^th^ decade of the life [4]. There is a slight male predominance, with a sex ratio of 1,6 [5, 6].

The clinical symptomatology is not specific; can be abdominal pain or mass [7–9], alteration in the general condition [10, 11], or signs related to a vascular or nervous compression [10] can all be experienced by the patient. Hepatic and pulmonary metastasis can be observed in the course of a leiomyosarcoma of the mesocolon. They represented the main causes of the death. Involvement of the nodes is rare [10]; ultrasonography and computer tomographic scanning of the abdomen are very helpful in view of the diagnosis of leiomyosarcoma. Leiomyosarcoma usually appears at ultrasonography as a high volume and irregular mass with multiples echogenic lobes, and central cavity of necrotic areas. Computer tomography scanning also exhibits a rounded in homogenous tissular mass with irregular edges, central necrotic areas and enhancement of the peripheral zone.

Pathological exam combined with immunohistochemical studies usually confirms the diagnosis of pleomorphous sarcomas are defined as mesenchymal tumors consisting on pleomorphic spindle- cells with different forms and size and the pleomorphic is high lightened by the nuclear atypical and the anyso caryopses [3].

Durant the years 1980 [3], a balanced proposition of pleomorphous sarcoma was classified as malignant histocytofibromas (MHF). Currently, the MHF is no way considered as a specific clinic pathologic entity but just as a morphological feature seen in many tumors, including melanomas, lymphomas and carcinomas. Because of the progress in immunohistochemistry and electronic microscopy, some criteria's have been included in order to make a subclassification of the pleomorphous sarcoma entity.

Macroscopically, pleomorphous sarcoma presents as a white to grey-pinked hard and firm great mass, sometimes elastic or renittent. More it is more than 10 cm, the tumor is generally lobulated with smooth areas corresponding to intratumoral necrotic zones, and recovered by a complex channel of blood vessels.

At the microscopic level, pleomorphous leiomyosarcoma contain pleomorphous cells, which are around and spindle shaped, extending as longs fasciles takings contact the ones to others in rights angles. Atypical cells are frequents because of the presence of giants cells, normal and abnormal mitosis. The cells have an abundant eosinophil and fibrillary cytoplasm. The nucleus muscle and desmine (50 to 70% of all the cases) [3].

The differential diagnosis is essentially with others clinical entities such as undifferenciated pleomorphous sarcoma, gastro-intestinal tumors of the stroma, pleomorphous rhabdomyosarcoma. Immunohistochemistry studies allow performing the diagnosis [3].

The benign or malignant nature of a pleomorphous sarcoma is not always easy to establish. Grading's system relying on histological parameters that have been described in order to better differentiate between the different tumoral grades and so the prognosis [12]. Like for the other sarcoma of the smooth tissues, prognostic factors on the histological type, the depth of the tumor, the high grade, the tumor size (more than 5cm), tumoral necrosis great mitotic activity, infiltration in the neighboring structures, vascular invasion and the localization other than a limb, recurrence. Generally pleomorphous leiomorphous are considered as very aggressive and metastatic pleomorphous sarcomas.

Moreover, it has been shown that myogenic differentiation was a deleterious prognostic factors [13]. The treatment of pleomorphous sarcomas rely on surgery. The excision of the tumor in a monobloc fashion, with the viscera and without any tumoral effraction, is the standard of therapy [7]. Nodes excision is not systematic because involvement or recurrences at these sites are rare [8]. The high recurrence rate after surgery has led to investigations into the use of combined modality treatment for these tumours. As the most common form of relapse in retroperitoneal sarcoma is a local recurrence, adjuvant radiotherapy has been proposed to enhance the cure rates and improve the relapse free survival. Studies in extremity sarcomas have demonstrated improved local control rates when surgery was combined with radiotherapy. Globally, the sensitivity of sarcomas to irradiation is low [14]. However, there is no consensus regarding use of neoadjuvant or adjuvant chemotherapy. Neo adjuvant chemotherapy (NACT) is given with an aim to improve the resectability of the tumour. The advantages of NACT are a reduction in the size of tumours, probability of a margin negative resection and determining the tumour response to chemotherapy. In addition, any micrometastatic disease could be controlled. The disadvantages are that a tumour may not respond to chemotherapy and a potentially resectable tumour may become inoperable. Chemotherapy may also worsen the performance status of the patient [14]. Several studies, most of them non-randomized have not shown any consistent benefit withNACT. In one large study, Meric et al,reported results of 65 patients of RPS with NACT doxorubicin or ifosfamide, of these 23 had resectable RPS [15].

After surgical treatment, the course of pleomorphous sarcoma is characterized by the high frequency of local recurrences, as up as 44% to 85% [7, 11]. The management of these recurrences relies on iterative surgeries. The global rate of survival at 5 years is about 20 to 30% [7, 11].

## Conclusion

Pleomorphous leiomysarcoma of the mesocolon is a rare tumor. The positive diagnosis of this condition can be made on pathological exam combined with immunohistochemistry studies after surgery. The association of an adjuvant therapy remains controversial; however, adjuvant chemotherapy can be suggested in the case of a bad prognosis, a short time after the surgical procedure.
